# Identification of the human cytomegalovirus gHgLgO trimer as the central player in virion infectivity

**DOI:** 10.1371/journal.ppat.1013341

**Published:** 2025-07-24

**Authors:** Lena Thiessen, Roberto Garuti, Lucie Kubic, Miwako Kösters, Divya Amarambedu Selvakumar, Thomas Krey, Irene Görzer, Thomas Fröhlich, Barbara Adler

**Affiliations:** 1 Max von Pettenkofer-Institute and Gene Center, Department of Virology, Faculty of Medicine, Ludwig-Maximilians-University (LMU) Munich, Munich, Germany; 2 German Center for Infection Research (DZIF), Partner Site Munich, Munich, Germany; 3 Laboratory for Functional Genome Analysis LAFUGA, Gene Center, Ludwig-Maximilians-University, Munich, Germany; 4 Center of Structural and Cell Biology in Medicine, Institute of Biochemistry, University of Lübeck, Lübeck, Germany; 5 German Center for Infection Research (DZIF), Partner Site Hamburg-Lübeck-Borstel-Riems, Lübeck, Germany; 6 Institute of Virology, Hannover Medical School, Hannover, Germany; 7 Excellence Cluster 2155 RESIST, Hannover Medical School, Hannover, Germany; 8 Centre for Structural Systems Biology (CSSB), Hamburg, Germany; 9 Center for Virology, Medical University of Vienna, Vienna, Austria; Florida State University, UNITED STATES OF AMERICA

## Abstract

Glycoproteins in the viral envelope of human cytomegalovirus (HCMV) orchestrate virion tethering, receptor recognition and fusion with cellular membranes. The glycoprotein gB acts as fusion protein. The gHgL complexes gHgLgO and gHgLpUL(128,130,131A) define the HCMV cell tropism. Studies with HCMV lacking gO had indicated that gHgLgO, independently of binding to its cellular receptor PDGFRα, plays an important second role in infection. Here, we identified a gO mutation which abolished virus particle infectivity by preventing the interaction of gHgLgO with host cell heparan sulfate proteoglycans (HSPGs). We could not only show that gHgLgO – HSPG interactions are a genuine second role of gHgLgO, but also that gHgLgO is a main player in determining the infectivity of HCMV virus particles. This challenges long-accepted textbook knowledge on the role of gB and gMgN complexes in virion tethering. Additionally, it adds the gHgLgO complex to the antigens of interest for future HCMV vaccines or treatments.

## Author summary

Life threatening diseases in immunocompromised individuals and severe damage of unborn children after intrauterine infection with HCMV strongly ask for a vaccine efficiently preventing HCMV disease. Numerous trials with vaccines predominantly targeting HCMV virion glycoproteins have been performed but none of them met the requirements for a license. The CMV gHgLgO glycoprotein complex has been shown to be crucial for virion infectivity and infection of first target cells *in vivo*, but also due to an incomplete understanding of the mechanisms determining its role in infection, it was never considered as a vaccine antigen. By using a highly specific HCMV gO mutant, we could here show that, besides binding the entry receptor PDGFRα, the gHgLgO complex determines particle infectivity by tethering virus particles to cell surface HSPGs. This role has for a long time been attributed to the glycoproteins gB and gM/gN. Our data challenge this. Complementation of vaccine antigens like gB or the gHgLpUL(128,130,131A) complex, which have already been used in vaccine trials, with the until now neglected gHgLgO complex might take the anti-HCMV vaccine development a step forward.

## Introduction

Infections with human cytomegalovirus (HCMV) are widespread in the human population and persist like all herpesviruses for life [1]. In immunocompromised individuals, infections can result in life-threatening diseases and infections of the fetus are a major cause of congenital birth defects [1]. Therefore, significant efforts have been made to develop vaccines for preventing or restricting infection mainly by using virion glycoprotein complexes as antigens [2–4]. Up to now, none of the vaccines has been highly efficient.

Infection of target cells by HCMV involves three steps: tethering to cell surfaces, receptor recognition and fusion with cellular membranes [5,6]. Several virion glycoprotein complexes are involved in these steps including the trimeric gHgLgO complex, the pentameric gHgLpUL(128,130,131A) complex and the gB homotrimer. The heterodimer gHgL and the gB trimer form the viral fusion machinery while the gHgL-associated proteins promote binding to host cell receptors [5,6]. Deletion of the gHgL-associated proteins of the pentamer results in restriction of the HCMV cell tropism [5]. In contrast, deletion of gO results in a more dramatic reduction of infectivity for all host cells [7–13]. The cellular entry receptor bound by gHgLgO is platelet-derived growth factor receptor alpha (PDGFRα) [11,12,14–16], yet loss of this interaction cannot explain the loss of infectivity for PDGFRα-negative cells. Several studies addressing impaired infectivity of HCMV mutants lacking gO had pointed towards a second role of gHgLgO in initiating cell infection [13,14,17,18]. When studying the role of the functionally homologous gHgLgO complex of murine cytomegalovirus (MCMV) *in vivo*, we have shown that deletion of gO nearly completely abolished susceptibility of mice to MCMV infection by preventing infection of different first target cells [19]. In contrast, deletion of the MCMV gHgLMCK2 complex, which is functionally homologous to the pentameric complex of HCMV, only resulted in a minor impairment of infection [19–22]. Thus, the gHgLgO complex might be an interesting vaccine target. Yet, for the development of vaccines targeting the HCMV trimer, a prerequisite would be to fully understand the role of gHgLgO in infection.

Here, we used an HCMV gO mutant in which five basic amino acids in the highly conserved peptide site 249 to 254 of gO were exchanged to alanines [13]. This mutant showed a strong and cell-type independent loss of infectivity but normal binding to PDGFRα. A profound analysis of this mutant revealed the postulated, but until now unidentified second role of gHgLgO in HCMV infection. We could show that tethering of HCMV particles to host cells is highly dependent on the interaction of the gHgLgO complex with heparan sulfate proteoglycans (HSPGs), a function whose loss goes along with the loss of particle infectivity. We could also show that complexes of gHgLgO and gB [14] do not contribute to HSPG binding. Complex formation of gHgLgO with gB could even block the intrinsic capacity of gB [23] to bind HSPGs.

## Results

### HCMV mutants revealing the dual role of glycoprotein gO

**Fig 1:**
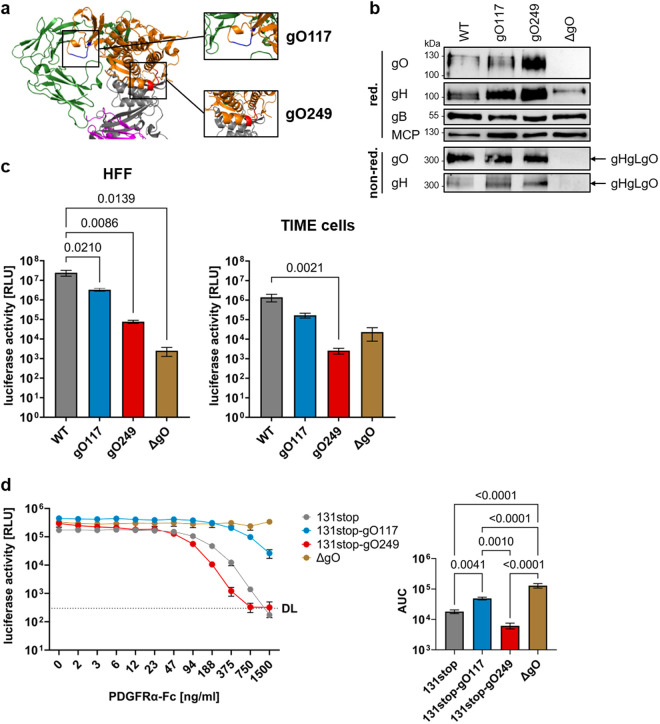
The gO249 mutation results in a PDGFRα-independent loss of particle infectivity. **(a)** Cryo EM structure [16] of gHgLgO bound to PDGFRα (gH (purple), gL (grey), gO (orange), PDGFRα (green); PDB: 7LBF) with locations of mutated sequences of gO117 and gO249 highlighted in blue and red, respectively. **(b)** Levels of virion glycoproteins gH, gO and gB were assessed in virion lysates by WB analysis under reducing (red.) conditions. The formation of gHgLgO complexes was assessed under non-reducing (non-red.) conditions. Levels of major capsid protein (MCP) reflect comparable numbers of virus particles. One representative experiment of three is shown. **(c)** HFF (left panel) or TIME cells (right panel) were infected with 100 virus particles/cell and infection was assessed by luciferase assay 48 h p.i. Shown are means + /- SEM of independent experiments. *P* values of statistically significant differences are depicted (HFF: one-way ANOVA, *n*(WT, gO117, gO249) = 4, *n*(ΔgO) = 3; TIME cells: Kruskal-Wallis test, *n*(WT) = 6, *n*(gO117, gO249) = 4, *n*(ΔgO) = 3). **(d)** Infection of HFF after preincubation of viruses with increasing concentrations of soluble recombinant PDGFRα-Fc. 48 h p.i., infection was assessed by luciferase assay. Shown are means + /- SEM of independent experiments. Right panel: Calculation of the area under the curve (AUC) of normalized data (one-way ANOVA, *n*(WT) = 6, *n*(gO117, gO249, ΔgO) = 3). *P* values of statistically significant differences are depicted. RLU: relative light units. DL: detection limit.

Deletion of the HCMV glycoprotein gO results in elimination of PDGFRα-dependent HCMV entry [11,12,14,24]. In addition, gO-negative HCMV particles show a dramatic reduction in infectivity for all host cells which is independent of PDGFRα [10,13,14,17]. So far, all attempts to understand this second role of gO were unsuccessful. We screened the literature for HCMV gO mutants, which exhibited a major loss of particle infectivity for different host cells, but were still capable of forming gHgLgO complexes in virions. A mutagenesis of conserved regions in gO described a mutant with the desired phenotype [13]. This mutant comprised a change of amino acids 249-**RK**L**KRK**-254 to 249-**AA**L**AAA**-254 and will here be called gO249. For a thorough characterization of gO249, we compared it with a second gO mutant with amino acids 117-**RK**PA**K**-121 changed to 117-**AA**PA**A**-121 [25], here called gO117. The gO117 mutant has been shown to be impaired in binding to PDGFRα. Based on recently published cryo EM structures of the trimer [16], the gO249 mutation could be localized proximal to glycoprotein gL and opposite to the PDGFRα binding site (Fig 1a). The gO117 mutation could be localized to the PDGFRα interface (Fig 1a). We introduced these mutations into TB40-BAC4-luc virus (here designated as WT virus) [8]. Western blot (WB) analysis of virus particle lysates revealed comparable contents of the virion glycoproteins gH, gO and gB for WT, gO249 and gO117 (Fig 1b, upper panels). Under non-reducing conditions, the quantities and electromobilities of gHgLgO complexes of gO249 and gO117 viruses were comparable to those of WT virus (Fig 1b, lower panels). For comparison, extracts of gO-negative virions (ΔgO mutant) [8] are shown (Fig 1b). To compare the infection capacities of WT, gO249, gO117 and ΔgO viruses for fibroblasts and endothelial cells, PDGFRα-positive human foreskin fibroblasts (HFF) and PDGFRα-negative immortalized microvascular endothelial cells (TIME cells) were chosen (S1a Fig). HCMV entry into TIME cells is strictly dependent on the pentameric complex [8,14,26], whereas entry into HFF predominantly depends on the trimeric gHgLgO complex [14]. Cells were infected with equal numbers of virus particles and infection capacities evaluated using a luciferase assay (Fig 1c). Consistent with its first characterization [13], gO249 showed strongly reduced infection capacities for fibroblasts (0.3% of WT) and endothelial cells (0.2% of WT). Compared with gO249, infection capacities of gO117 were less reduced (HFF: 13.4% of WT; TIME cells: 12% of WT). Infection capacities of the ΔgO mutant showed a reduction to 0.01% on HFF and 1.7% on TIME cells when compared to WT virus. The stronger reduction of ΔgO compared to gO249 on HFF may reflect the loss of additional gO functions, whereas the reduced attenuation on TIME cells might reflect elevated levels of pentameric complex in gO-negative virions as described before [14,27–29].

The differences in infection capacities also shaped the growth curves of gO mutants (S1b Fig). HFF were infected with equal infectious doses of WT, gO249, gO117 or ΔgO virus and virus growth was monitored by determining infectious virus in cell culture supernatants. Particle numbers and specific infectivity, exemplarily determined for day eight supernatants, confirmed that the gO249 mutation or deletion of gO impaired particle infectivity, but not particle release. The reduced particle infectivity became apparent in the growth curves by a strongly reduced production of infectious virus.

To find out whether the gO249 mutation impairs binding to PDGFRα, we performed a quantitative Fc pulldown assay [30]. We used lysates of pentamer-negative virus particles (TB40-BAC4-luc-131stop virus [8], here called 131stop virus) as source of gHgLgO complexes. Like pentamer-positive viruses, 131stop viruses express comparable amounts of gHgLgO_WT_ and gHgLgO_249_ (S2a Fig). The lysates for the Fc pulldown assay were adjusted for equal amounts of gO by quantitative WB (S2b Fig) and co-incubated with increasing amounts of recombinant PDGFRα-Fc bound to Protein A sepharose beads. By precipitating the beads, gHgLgO complexes bound to PDGFRα-Fc were separated from non-bound gHgLgO complexes. We could show that PDGFRα-Fc precipitated comparable amounts of gO and gO249 proteins resulting in comparable binding curves for gHgLgO_WT_ and gHgLgO_249_ (S2c Fig). Comparable binding of gHgLgO_WT_ and gHgLgO_249_ to PDGFRα could also be confirmed by immunoprecipitation experiments using PDGFRα-Fc to precipitate the complexes from lysates of HEK293T cells transfected with plasmids expressing gH, gL, wildtype gO or gO249 (S2d Fig).

To evaluate the role of the gHgLgO – PDGFRα interactions during infection, we infected HFF with WT, gO249, gO117 and ΔgO viruses pre-incubated with increasing concentrations of soluble recombinant PDGFRα-Fc protein. Due to the different infection efficiencies of the mutants, the virus inocula were adjusted such that infection resulted in equal numbers of infected cells 48 h p.i., here reflected by comparable luciferase signals. With the exception of ΔgO, the 131stop genomic background was chosen to restrict infection of WT, gO249 and gO117 viruses to gHgLgO-driven entry. As shown before [14], 131stop virus could be completely inhibited with high concentrations of recombinant PDGFRα protein, whereas the pentamer-dependent infection with ΔgO virus could not be inhibited at all (Fig 1d). The 131stop viruses expressing wildtype gO or gO249 were inhibited to a similar extent which suggested that the gO249 mutation does not interfere with PDGFRα-binding. In contrast, gO117 virus was inhibited to a much lesser extent.

### gHgLgO shapes HCMV particle infectivity by promoting attachment to host cells

**Fig 2:**
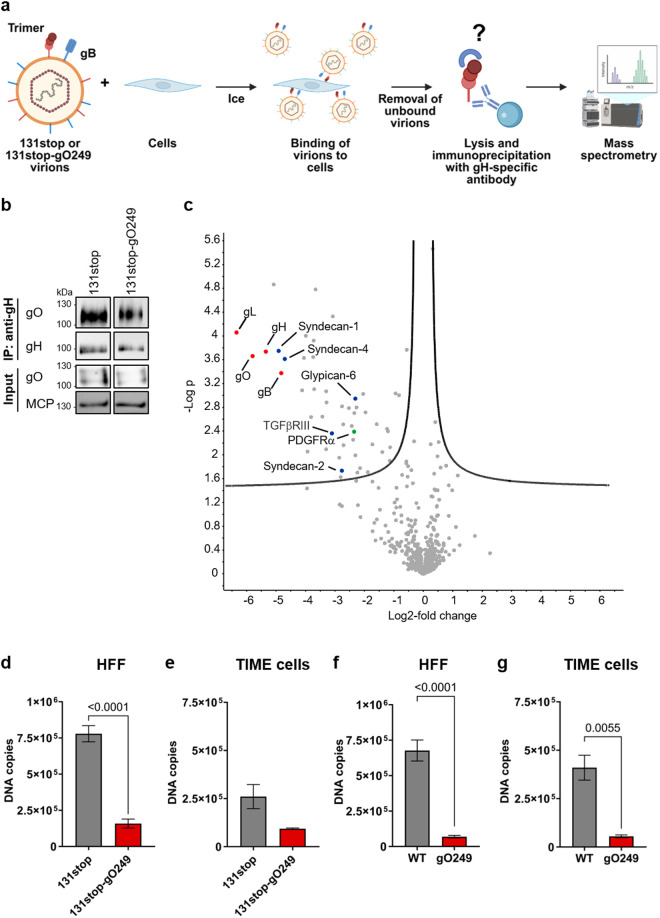
Screening for gHgLgO interaction partners reveals decreased binding of the gO249 mutant to cell surfaces. **(a)** Schematic presentation of experiments performed to identify cell surface proteins which interact with gHgLgO_WT_ or gHgLgO_249_. 131stop or 131stop-gO249 virions were co-incubated with cells for 1 h on ice. Unbound virions were removed and cells were lysed subsequently. An immunoprecipitation was performed using the gH-specific antibody 14-4b followed by LC-MS/MS analysis to identify protein interaction partners of gH for 131stop and 131stop-gO249 viruses. Created in BioRender. Thiessen, L. (2025) https://BioRender.com/a71z766. **(b)** WB analysis of gH and gO immunoprecipitated from lysates of 131stop and 131stop-gO249 virions using the gH-specific antibody 14-4b. One representative experiment of four is shown. **(c)** Volcano plot of LC-MS/MS data from anti-gH immunoprecipitates of lysates of HFF co-incubated with 131stop or 131stop-gO249 virions as described under **(a)**. Data from 131stop-gO249 virions were compared to 131stop virions and depicted as –Log *p*-value versus Log2-fold change. HCMV glycoproteins are highlighted in red, the gHgLgO receptor PDGFRα in green, TGFβRIII and syndecans and glypican in blue. Data are derived from three independent experiments. **(d-g)** HFF (*n*(**d**) = 5, *n*(**f**) = 6) or TIME cells (*n* (**e and g**) = 3) were incubated with 5x10^7^ virus particles. Bound virus particles were quantified by qPCR. Shown are means + /- SEM of independent experiments. *P* values of statistically significant differences are depicted (Student’s t-test or Mann-Whitney test).

Several studies had suggested or shown that gHgLgO is crucial for particle infectivity independent of PDGFRα and the host cell [10,13,17,18]. The gO249 mutant has lost most of its particle infectivity without a change in PDGFRα-binding. This phenotype implied a widespread second cell-surface (co-)receptor for gHgLgO. Therefore, we searched for surface proteins of HFF which can bind virions containing gHgLgO_WT_ complexes, but not virions containing gHgLgO_249_ complexes. We used 131stop and 131stop-gO249 viruses to exclude interactions of surface proteins with the pentameric complex. We co-incubated HFF with equal numbers of virus particles, lysed cells and viruses bound to cell surfaces and precipitated gHgLgO complexes from these lysates using a gH-specific antibody followed by mass spectrometry (LC-MS/MS) of the precipitated proteins (Fig 2a). The anti-gH antibody used equally precipitated gHgLgO_WT_ and gHgLgO_249_ complexes (Fig 2b). When comparing proteins co-precipitated from HFF-bound 131stop or 131stop-gO249 virions, 51 hits were identified which were significantly lower for the 131stop-gO249 mutant (Fig 2c and S1 Table). These hits included known HCMV particle-associated proteins like exosome-associated proteins [31], the gHgLgO cellular receptor PDGFRα, the previously identified gHgLgO-binding protein TGFβRIII (also known as betaglycan) [16] and cell surface heparan sulfate proteoglycans (HSPGs) like syndecans and glypican. Surprisingly we additionally observed a strongly reduced precipitation of gH, gL, gO and the gHgLgO-associated glycoprotein gB [14]. This indicated that the gO249 mutation not just simply abolished interaction with specific gHgLgO-binding proteins, but generally reduced the binding of virions to HFF. As the 51 hits identified for HFF might include PDGFRα-dependent hits, we repeated the LC-MS/MS analyses using PDGFRα-negative TIME cells. When comparing proteins co-precipitated from TIME cell-bound 131stop or 131stop-gO249 virions, we could identify 13 hits which were significantly less abundant for the 131stop-gO249 mutant. These hits again included gH, gL and gO (S3 Fig and S2 and S3 Tables) which suggested that the gO249 mutation also reduced binding of virions to TIME cells. Besides gH, gL and gO, the most striking overlap in gHgLgO-binding proteins for 131stop virus on HFF and TIME cells was TGFβRIII.

To address whether the mass spectrometry data reflect differences in virion binding to cell surfaces, we repeated the experiments, but this time quantified virions bound to cell surfaces using qPCR. Binding of 131stop-gO249 virions to HFF was strongly reduced when compared to 131stop virions (Fig 2d) which confirmed that gO249 virions exhibit a defect in cell attachment. As binding of 131stop virions to TIME cells was relatively weak, the difference between binding of 131stop and 131stop-gO249 virions to TIME cells was statistically not significant (Fig 2e). A comparison of pentamer-positive WT and gO249 viruses exhibited clear differences in binding both for HFF and TIME cells (Fig 2f and 2g). In summary, these data suggest that gHgLgO strongly contributes to virion tethering through a PDGFRα-independent interaction with host cell surfaces.

### gHgLgO—HSPG interactions are important determinants of HCMV particle infectivity

**Fig 3:**
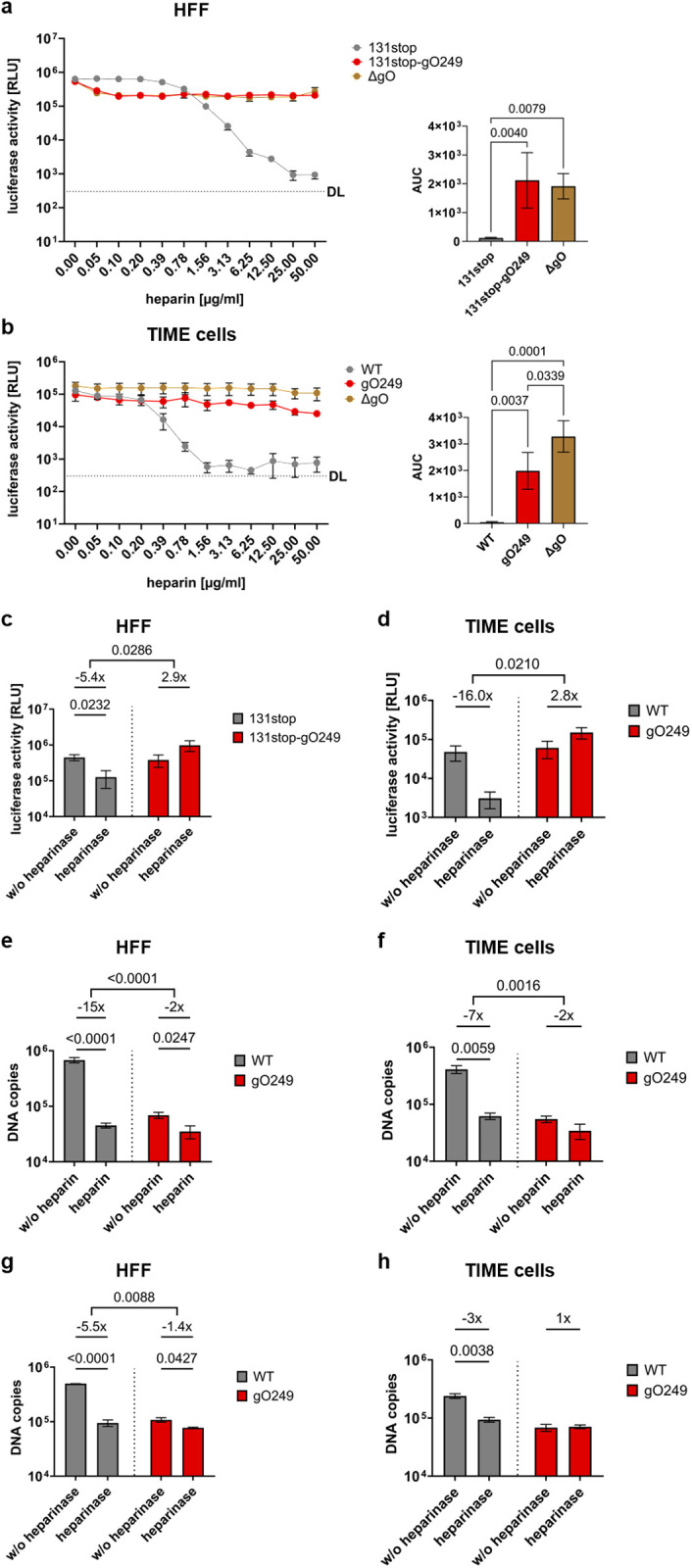
Infection and virion attachment require interaction of gHgLgO with HSPGs. **(a)** HFF and **(b)** TIME cells were infected with 131stop, 131stop-gO249 or ΔgO virus in the presence of increasing concentrations of heparin. The virus inocula were adjusted such that infection resulted in comparable luciferase signals. 48 h p.i., infection was assessed by luciferase assay. Shown are means + /- SEM of three independent experiments. Right panels: Calculation of the AUC of normalized data (one-way ANOVA). **(c)** HFF (**n* *= 4) or **(d)** TIME cells (**n* *= 3) were pre-treated with heparinase I, II and III followed by infection with 131stop or 131stop-gO249 virus (HFF) or WT or gO249 virus (TIME cells). 48 h p.i., infection was assessed by luciferase activity. Shown are means + /- SEM of independent experiments. Statistical significance was determined for pairwise comparisons of heparinase-treated and untreated cells (Student’s t-test or Mann-Whitney test). Additionally, fold changes of pairwise comparisons were calculated and analyzed (Student’s t-test or Mann-Whitney test). **(e and g)** HFF or **(f and h)** TIME cells were incubated with 5x10^7^ WT or gO249 virus particles in the presence (100 µg/ml) or absence of heparin (**e** and **f**) or after pre-treatment of the cells with heparinase I, II and III (**g** and **h**) and virus particles bound to cells were quantified by qPCR. Shown are means + /- SEM of independent experiments (*n*(**e**) = 6, *n*(**f-h**) = 3). Statistical significance was determined for pairwise comparisons of treated and untreated infections (Student’s t-test). Additionally, fold changes of pairwise comparisons were calculated and analyzed (Student’s t-test and Mann-Whitney test). The data showing virus binding in the absence of heparin (**e** and **f**) are identical to the data of Fig 2f and 2g. **(a-h)** *P* values of statistically significant differences are depicted. DL: detection limit.

The strongly reduced infectivity and binding capacity of gO249 virus particles suggested that the gO249 mutation abolished the interaction of virions with cell surface molecules promoting virus attachment. Interestingly, syndecans, glypican and also TGFβRIII [32], which were co-precipitated with gHgLgO, are heparan sulfate proteoglycans (HSPGs). Adsorption of virus particles to cell surface HSPGs is an accepted first step in herpesvirus and HCMV infection [33,34]. The gO249 mutation (249-**RK**L**KRK**-254 to 249-**AA**L**AAA**-254) reverted a linear sequence of basic amino acids, a motif characteristic for HSPG-binding proteins [35], into a non-polar amino acid sequence. Therefore, we hypothesized that gHgLgO may directly or indirectly interact with HSPGs and that the reduced cell surface binding of gO249 may be due to an impaired interaction of gHgLgO_249_ with HSPGs. To address this, we infected HFF and TIME cells with equal infectious doses of viruses expressing wildtype gO or gO249 and for comparison also with ΔgO virus in the presence of increasing concentrations of the HSPG mimic heparin. While viruses expressing wildtype gO could be inhibited in a concentration-dependent manner, both on HFF and TIME cells, infections with gO249 and ΔgO viruses were resistant to heparin (Fig 3a and 3b). This indicated that gO and more specifically amino acids 249 to 254 of gO promote the virion - HSPG interaction. HSPG-dependence of viruses expressing wildtype gO and no HSPG-dependence of gO249 viruses could also be observed when host cells were pre-treated with heparinase (Fig 3c and 3d).

Differences in HSPG interactions between WT and gO249 viruses should also become apparent when binding to cells is studied. Binding of WT virus to HFF and TIME cells was clearly inhibited in the presence of heparin (Fig 3e and 3f) and by heparinase pre-treatment of the host cells (Fig 3g and 3h). Binding of gO249 virus could not much further be inhibited (Fig 3e to 3h). Thus, the 249 mutation of gO strongly impaired HSPG-dependent tethering of virions which provides a highly plausible explanation for the low infectivity of gO249 virus particles.

**Fig 4:**
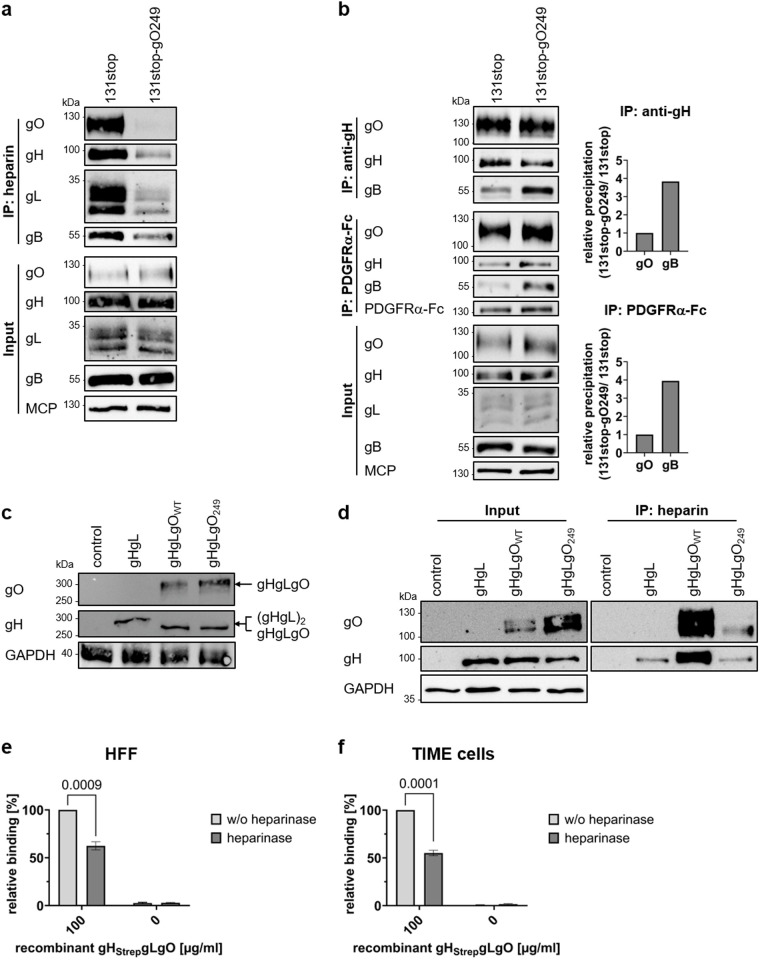
gHgLgO is sufficient to bind to HSPGs and mutation of amino acids 249 to 254 of gO abolishes this interaction. (**a**) WB analysis of gH, gL, gO and gB precipitated from 131stop and 131stop-gO249 virion lysates using heparin agarose. One representative experiment of four is shown. (**b**) WB analysis of gH, gL, gO and gB precipitated from 131stop and 131stop-gO249 virion lysates using an anti-gH antibody (14-4b) or soluble recombinant PDGFRα-Fc. Representative experiments of three are shown. Right panels: ImageJ quantification of gO and gB WB signals. gO precipitation levels were normalized and relative gB levels determined. (**c**) WB analysis of gH and gO expression in lysates of HEK293T cells transfected with plasmids expressing GFP (control), gH and gL (gHgL), gH, gL and wildtype gO (gHgLgO_WT_) or gH, gL and gO249 (gHgLgO_249_). Lysates were prepared under non-reducing conditions. GAPDH levels served as loading control. One experiment of two is shown. (**d**) WB analysis of gH and gO precipitated from lysates of HEK293T cells expressing GFP (control), gHgL, gHgLgO_WT_ or gHgLgO_249_ using heparin agarose. GAPDH levels served as loading control. One experiment of two is shown. (**e**) HFF and (**f**) TIME cells were pre-treated with heparinase I, II and III followed by incubation with recombinant gH_Strep_gLgO_WT_ (100 µg/ml). Binding of recombinant protein to cells was assessed using an ELISA detecting Strep-tagged proteins. Binding of recombinant gH_Strep_gLgO_WT_ to untreated cells was arbitrarily set to 100%. Shown are means + /- SEM of three independent experiments. Statistical significance was determined by pairwise comparison of heparinase-treated and -untreated cells (Student’s t-test). *P* values of statistically significant differences are depicted.

We also compared binding of gHgLgO_WT_ and gHgLgO_249_ complexes to heparin by precipitating gHgLgO from 131stop and 131stop-gO249 virion lysates using heparin agarose (Fig 4a). Precipitation of gHgLgO_249_ was strongly reduced and went along with reduced precipitation of gB from 131stop-gO249 virion lysates. Thus, very likely, virion gB alone does not efficiently bind to heparin, but is co-precipitated as it forms a complex with gHgLgO [14]. This raised the question whether gHgLgO - gB complexes are required for HSPG binding and whether the impaired binding of gHgLgO_249_ to heparin reflects the loss of gHgLgO - gB complex formation. To address the latter point, we compared the interaction of gB with gHgLgO_WT_ and gHgLgO_249_ by co-precipitating gH, gO and gB from virion lysates using recombinant PDGFRα-Fc or a gH-specific antibody. The interaction of gHgLgO_249_ with gB was not reduced, but even 4-fold stronger than the interaction of gHgLgO_WT_ and gB (Fig 4b). Yet, we could not exclude that the gHgLgO_249_ – gB complex is specifically impaired in binding to HSPG. To exclude that gHgLgO needs gB to bind to HSPG, we expressed recombinant gH, gL and gO in HEK293T cells. Both, wildtype gO and gO249 formed gHgLgO complexes in transfected cells (Fig 4c). WB analyses of lysates of infected HFF showed two distinct bands for wildtype gO and gO249 of whom only the upper bands could be detected in lysed virions (S4 Fig). These double bands were also detectable in transfected cells (Fig 4d, left panel). When using heparin agarose to precipitate the recombinant gHgLgO complexes, we found that the upper gO band could only be precipitated from cells expressing recombinant gHgLgO_WT_ and not from cells expressing gHgLgO_249_ (Fig 4d, right panel). This indicated that the gHgLgO complex alone is sufficient to bind to heparin and that the gO249 mutation abolishes this interaction. Heparin agarose also precipitated gH from lysates of cells transfected with gH and gL alone or with gH, gL and gO249, although to a much lower extent. This may be an artefact of recombinant gHgL complexes, yet, it cannot completely be excluded that also moieties of gHgL complexes can to some degree interact with HSPGs.

To confirm the interaction of gHgLgO with HSPGs in a more physiological setting, namely on the surface of HCMV host cells, we co-incubated heparinase-treated and untreated HFF and TIME cells with soluble recombinant gH_Strep_gLgO complex and measured binding of this complex using an ELISA detecting the Strep-Tag of gH. As previously described for other recombinant gHgLgO complexes [17], the gH_Strep_gLgO complex could block HCMV infection of HFF and TIME cells (S5 Fig). In the ELISA we could confirm binding of gH_Strep_gLgO to HFF and TIME cell surfaces and we observed a significant reduction of binding when cells were pre-treated with heparinase (Fig 4e and 4f). Thus, attachment of gHgLgO to cell surfaces is dependent on an interaction with HSPG and is not restricted to cells expressing PDGFRα.

We could hardly precipitate gHgLgO_249_ complexes using heparin, but still observed residual gB precipitation from gO249 virion lysates and residual inhibition of gO249 binding. Therefore, we wondered whether ΔgO virions lacking gHgLgO complexes show a complete loss of gB precipitation by heparin agarose. Interestingly, gB could be precipitated from ΔgO virions indicating a gB - heparin interaction independent of gHgLgO (S6a Fig). When comparing virion binding of WT, gO249 and ΔgO viruses to HFF and TIME cells, we observed an enhanced binding of ΔgO virions compared to gO249 virions which could be completely inhibited by heparin (S6b and S6c Fig). In summary, these data might be interpreted such that gHgLgO is an important driver of virion attachment by binding to HSPGs. gB can also bind HSPGs, although to a much lesser extent. The capacity of gB to bind HSPGs becomes particularly apparent in ΔgO infections. The gO249 mutation not only results in a loss of the gHgLgO - HSPG interaction, but very likely additionally blocks binding of gB to HSPGs by trapping gB in tight gHgLgO_249_ - gB complexes.

### gHgLgO—From tethering to cell surface HSPGs to PDGFRα docking

**Fig 5:**
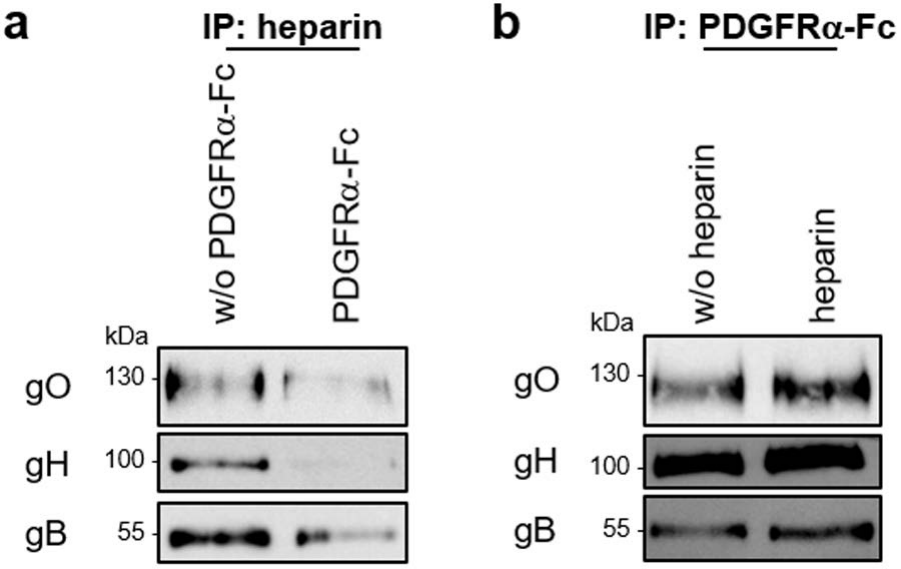
Competitive binding of heparin and PDGFRα to gHgLgO suggests overlapping binding sites. gHgLgO complexes were precipitated from lysates of 131stop virions using (**a**) heparin agarose or (**b**) soluble recombinant PDGFRα-Fc. **(a)** Proteins bound to heparin agarose beads were then co-incubated with PDGFRα-Fc (4 µg/ml) for 4 h at 4°C. **(b)** Proteins bound to PDGFRα-Fc/Protein A sepharose beads were co-incubated with heparin (500 µg/ml) for 4 h at 4°C. **(a, b)** Afterwards, beads were washed, incubated with 2x sample buffer and analyzed by WB for gH, gO and gB. One experiment of two is shown.

**Fig 6:**
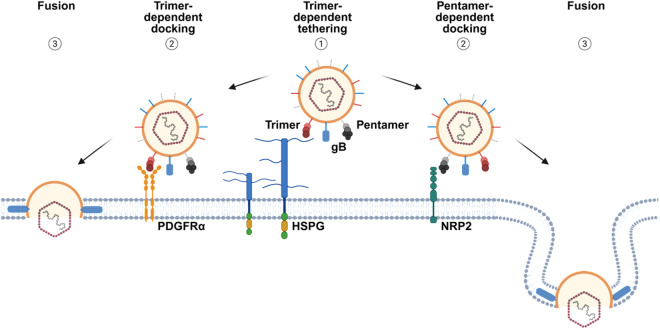
A model for the role of the HCMV trimer in infection of cells with free virus. In a first step, trimer – HSPG interactions tether virus particles to cell surfaces (1). gHgLgO-specific tethering is followed by docking of trimer or pentamer to their specific host cell entry receptors (2) and fusion of the viral envelope with cellular membranes (3). Exemplarily, the trimer-specific receptor PDGFRα and the pentamer-specific receptor NRP2 are shown. Created in BioRender. Thiessen, L. (2025) https://BioRender.com/j45u530.

Infectivity of cell free HCMV is shaped by binding of the gHgLgO trimer to host cell surface HSPGs and docking of the trimer or pentamer to HCMV entry receptors like PDGFRα or NRP2 [36], respectively. Assuming that gHgLgO binding to HSPGs is the first step in cell surface binding, this interaction with a relatively abundant cell surface molecule [33] either has to leave room for an additional interaction with the entry receptor PDGFRα or, if binding is overlapping, PDGFRα-interactions have to successfully compete with HSPG interactions. We tested this by consecutive addition of heparin and PDGFRα in immunoprecipitation experiments using virion lysates. Addition of recombinant PDGFRα-Fc to heparin agarose beads released most of gHgLgO and gB bound to the beads (Fig 5a). In contrast, gHgLgO and gB bound to PDGFRα-Fc on sepharose beads could not be removed by addition of heparin (Fig 5b). This indicated that binding to HSPGs and PDGFRα is competitive and that binding of gHgLgO to PDGFRα is stronger than binding of gHgLgO to heparin. We suggest a model for cell free infection in which gHgLgO determines efficient tethering of virus particles to cell surfaces followed by firm docking to entry receptors (Fig 6).

## Discussion

Cytomegaloviruses express two alternative gHgL glycoprotein complexes, gHgLgO and gHgLpUL(128,130,131A) which promote receptor recognition. For both, several host cell proteins have been described as binding partners and entry receptors [16,36,37]. HCMV can still infect cells when only one of the alternative gHgL complexes is deleted, yet the outcomes of deletion of the trimer or the pentamer are completely different. In cell culture, deletion of the pentamer abolishes infection of cell types like endothelial and epithelial cells, macrophages and dendritic cells, but for example not of fibroblasts [38]. In contrast, deletion of the trimer results in a drastic loss of infectivity for all host cells [9,10]. The gHgLgO complex uses PDGFRα as an entry receptor, an interaction which is resolved on a structural basis and also on the level of its role in infection [11,14,16]. Yet, the loss of the gHgLgO - PDGFRα interaction could not explain the cell type-independent loss of infectivity when gO is deleted.

Two recent studies had shown that soluble gHgLgO and also gO peptides bind to cell surfaces of PDGFRα-positive and -negative cells and interfere with HCMV infection [17,18]. They postulated additional widespread cell surface receptor(s) promoting a gHgLgO-dependent initial attachment to cells followed by entry receptor docking. Interestingly, their experiments, which were based on staining cell surface-bound trimer, excluded an involvement of heparin sulfate moieties [17].

Instead of comparing binding of recombinant gHgLgO complexes or comparing gO-positive or -negative virions, we wanted to identify these postulated receptor(s) by studying gO mutants with a general loss of virus infectivity, but not PDGFRα binding. A common strategy to identify mutants of interest is a protein-wide mutagenesis. Recently, a mutagenesis of conserved sites of gO has been published [13]. From this publication, we chose the gO249 mutant with amino acid sequence 249- **RK**L**KRK** -254 mutated to 249-**AA**L**AAA**-254. The gO249 mutant was still able to form virion gHgLgO complexes, but showed a drastic loss of infectivity for PDGFRα-positive and -negative cells, similar to gO-negative HCMV. Here, we could complement the initial characterization [13] by showing that virion gHgLgO_249_ interacts with PDGFRα like virion gHgLgO_WT_. This is consistent with findings for the respective recombinant gHgLgO complexes derived from HCMV strain AD169 [18]. We chose a quantitative mass spectrometry approach to identify cell surface receptor(s) on HFF and TIME cells bound by gHgLgO_WT_ but not by gHgLgO_249_ complexes. We made two important findings. First, gHgLgO_WT_ complexes clearly interact with HSPGs like syndecans, glypican and TGFβRIII and second, mutation of the 249- **RK**L**KRK** -254 site of gO abolished attachment of virions to cell surfaces. As virion - HSPG interactions are known for promoting infectivity of many viruses including HCMV, we followed up these findings by comparing (i) HSPG-dependence of WT and gO249 infections and (ii) interactions of gHgLgO_WT_ and gHgLgO_249_ complexes with heparin and sensitivity of these interactions to heparinase. This way, we could identify the gHgLgO complex as an important player in HSPG-driven tethering of virions to cells and show that the low infectivity of the gO249 mutant was due to a loss of virion – HSPG interactions. The gO249 mutation comprises a linear sequence of basic amino acids, typical for HSPG-binding proteins [35] and also described for other HSPG-binding herpesvirus glycoproteins like HSV-1 gB [39]. Our findings matched recent studies showing that preincubation of HCMV host cells with gO peptides comprising the 249 to 254 amino acid sequence and pre-incubation of virions with antibodies directed against this peptide blocked infection [18]. Interestingly, TGFβRIII was the only HSPG identified both on HFF and on TIME cells. TGFβRIII is a known binding partner of gHgLgO [36] and the gHgLgO - TGFβRIII complex has been characterized also on a structural basis [16]. TGFβRIII was excluded as an entry receptor [36], but up to now no function was assigned to the gHgLgO - TGFβRIII complex. Our findings might hint towards a role in virion tethering.

For many years, HCMV gB and gMgN were considered to promote initial virion attachment by interacting with cell surface HSPGs. This was mainly based on experiments which studied binding of purified gB and gMgN glycoproteins to heparin columns [23,40]. Yet, infection studies using a gB deletion mutant [41] or gN-truncated HCMV [42] had already questioned a major role of gB or gMgN in attachment to host cells. Interestingly, HCMV glycoproteins gH, gL, gO and gB co-expressed in transfected cells have already been shown to promote cell fusion by binding 3-*O*-sulfated heparan sulfate, yet, without revealing the exact heparan sulfate-binding partners [43]. We have shown before that gHgLgO and gB do form complexes in virions [14] which raised the question whether gHgLgO alone or gHgLgO - gB complexes promote HSPG- binding. Here, we could demonstrate that gHgLgO can strongly bind heparin independently of gB and that the gO249 mutation abolishes this interaction. Still, it remains unsolved what are the contributions of gHgLgO and gB to HSPG-binding and virion tethering. Our set of experiments comparing the heparin dependence of WT, gO249 and ΔgO virion binding could be interpreted in a way that in the absence of gO, gB can take over HSPG-binding, but not to the level exerted by gHgLgO. If gB is bound in strong complexes with gHgLgO_249_, it cannot compensate for the loss of gHgLgO-dependent tethering. This might be interpreted such that gB complexed with mutated and very likely also unmutated gHgLgO is unable to interact with HSPGs. As the gHgLgO content of virions varies between different HCMV strains [28], the relative contributions of gB and gHgLgO to virion infectivity may also vary.

All experiments presented here show that gHgLgO-dependent attachment to HSPGs is crucial for cell free infection. Yet, it has been shown by us and others that ΔgO mutants show efficient focal spread [9,14] which is also true for the gO249 mutant [13]. This discrepancy has also been observed *in vivo* in the MCMV infection of mice [19]. Deletion of the MCMV gHgLgO complex abolished infection of mice by preventing infection of first target cells after intravenous application of virus. In contrast, focal spread in for example livers could equally be secured by gHgLgO or the alternative complex gHgLMCK2.

In summary, trimer-dependent tethering of HCMV to HSPGs is an important driver of cell-free infection in cell culture and supposedly also of infection of first target cells *in vivo.* Trimer-dependent tethering is followed by firm docking of trimer or pentamer to their respective entry receptors. Thus, both, tethering and entry receptor binding in concert secure establishment of CMV infections. This suggests that complementation of gB and/or pentamer vaccines with trimer vaccines might be an option to design more efficient HCMV vaccines. For that, future research will have to expand recent studies [44] on important antibody target sites within the gHgLgO complex.

## Materials and methods

### Cells and viruses

Primary human foreskin fibroblasts (HFF; PromoCell) and HEK293T cells (ATCC: CRL-3216) were maintained in DMEM (Gibco) supplemented with 10% FCS and penicillin/streptomycin. Telomerase-immortalized human microvascular endothelial cells (TIME cells) [45] were cultured in Endothelial Cell Growth Medium MV 2 (PromoCell). All viruses used are derived from HCMV strain TB40/E cloned as a bacterial artificial chromosome (TB40-BAC4) [46] and express a firefly luciferase reporter (TB40-BAC4-luc virus) [8]. TB40-BAC4-luc-131stop virus (131stop) [8] lacks the gHgLpUL(128,130,131A) complex and TB40-BAC4-luc-ΔgO virus (ΔgO) [8] lacks the gHgLgO complex.

### Antibodies, recombinant proteins and reagents

Antibodies specific for HCMV were mouse anti-MCP, human anti-gB SM5–1, mouse anti-gH SA4 (all kindly provided by M. Mach, University Erlangen-Nürnberg, Germany), mouse anti-gH 14-4b (kindly provided by W. Britt, University of Alabama, Birmingham, USA), mouse anti-gO [47], mouse anti-UL128 4B10 (kindly provided by T. Shenk, University of Princeton, USA), mouse anti-gB 2F12 (Biozol) and rabbit anti-gL LS-C371267 (LSBio). Antibodies specific for cellular proteins or protein tags were rabbit anti-PDGFRα (Cell Signaling Technology), mouse anti-GAPDH GA1R (Thermo Fisher Scientific) and mouse anti-StrepMAB-Classic (IBA Lifesciences GmbH). Secondary antibodies were peroxidase-coupled goat anti-mouse (Sigma), kappa light chain-specific goat anti-mouse (Jackson ImmunoResearch), goat anti-rabbit (Dianova) and goat anti-human (Jackson ImmunoResearch). Soluble recombinant human PDGFRα-Fc fusion protein (R&D Systems) and heparin sodium salt (Sigma) were used for competition experiments.

### Expression and purification of recombinant HCMV gH_Strep_gLgO_WT_

Synthetic genes encoding HCMV gH, gL and gO (Twist Biosciences) were cloned into an insect cell expression vector described previously [48]. To allow for efficient purification, a double Strep-tag was fused to the C-terminus of gH together with an enterokinase (EK) cleavage site facilitating controlled proteolytic cleavage and co-transfected in equimolar ratio into Drosophila S2 cells. To initiate soluble protein expression, the stable cell line was scaled up and induced with 4 μM CdCl_2_ at a cell density of 6 × 10^6^ cells/mL. 5 days after induction, cells were harvested by centrifugation, and soluble proteins were purified from the supernatant using affinity chromatography on a Strep-Tactin XT 4Flow column (IBA Lifesciences), followed by size-exclusion chromatography using a Superose 6 Increase column (GE Healthcare) equilibrated in PBS. Protein was concentrated to approximately 3.9 mg/ml.

### BAC mutagenesis and virus reconstitution

Charge cluster to alanine (CCTA) mutants gO117 and gO249 were cloned based on a two-step mediated red recombination method [49]. The CCTA mutants were introduced into TB40-BAC4-luc and TB40-BAC4-luc-131stop. Specifically, for the gO117 mutant amino acids **RK**PA**K** at position 117 to 121 were changed to **AA**PA**A** using the primers gO117 forward (5’-TAACCTATCTGTGGTTCGATTTTTATAGTACCCAGCTT**GCTGC**ACCCGCC**GC**ATACGTCTACTCACAGTACAGGATGACGACGATAAGTAGGG-3’) and gO117 reverse (5’-ATCGTTTTAGCCGTATGATTGTACTGTGAGTAGACGTAT**GC**GGCGGGT**GCAGC**AAGCTGGGTACTATAAAACAACCAATTAACCAATTCTGATTAG-3’) and for the gO249 mutant the amino acids **RK**L**KRK** at position 249 to 254 were changed to **AA**L**AAA** using the primers gO249 forward (5’-CCCCAAGTATATTAACGGCACCAAGTTGAAAAACACTATG**GCTGC**ACTA**GC**A**GC**T**GC**ACAAGCGCCAGTCAAAGAACAAGGATGACGACGATAAGTAGGG-3’) and gO249 reverse (5’-TTTTAGTCTTTTTTTCTAATTGTTCTTTGACTGGCGCTTGT**GC**A**GC**T**GC**TAGT**GCAGC**CATAGTGTTTTTCAACTTGGCAACCAATTAACCAATTCTGATTAG-3’). Successful mutagenesis was confirmed by restriction fragment analysis and sequencing. To reconstitute BAC DNA to infectious virus, HFF were transfected with 1.5 µg purified DNA using FuGENE HD transfection reagent (Roche) according to the manufacturer’s protocol. Transfected cells were maintained until a strong cytopathic effect (CPE) became visible. To prepare cell-free virus, supernatants were precleared for 15 min at 2600 x g.

### Infections, virus stock preparation, virus titration and growth curves

For infection, medium of 90% confluent HFF or TIME cells was replaced by virus diluted in DMEM containing 5% FCS followed by a centrifugation step for 30 min at 860 x g at room temperature (RT) and an incubation for 1.5 h at 37°C.

For virus stock production, supernatants from infected HFF showing complete CPE were collected and precleared for 15 min at 2600 x g. After preclear, supernatant virus was pelleted by ultracentrifugation for 70 min at 37.700 x g at 4°C, the virus pellets resuspended in DMEM with 5% FCS or VSB buffer (50 mM Tris-HCl, 12 mM KCl, 5 mM EDTA, pH 7.8) and the concentrated stocks stored at -80°C.

Virus titers were determined by a TCID50 assay on HFF.

Multistep growth curves on HFF were performed on 24-well plates. For comparison of different virus mutants, cells were infected such that 24 h post infection, the percentage of initially infected cells was comparable for all viruses to be analyzed. Every second day, the medium was exchanged, the collected supernatants precleared and the virus in supernatants titrated or quantified using qPCR.

### Plasmids and transfection

HCMV glycoproteins expressed from eukaryotic expression vectors are based on the TB40-BAC4 sequence [46]. Full-length ORFs of gH, gL and gB were cloned in the pCR3 vector (Invitrogen). Wildtype gO and gO_249_ were cloned in the pFUSE-mIgG2B-Fc2 vector (InvivoGen). For that, the N-terminal signal peptides (amino acids 1–29) of gO_WT_ or gO_249_ were exchanged for the 20 amino acids of the human IL2 signal sequence and the Fc sequence of the vector was deleted. The IL2-gO fusion proteins are thus comprised of amino acids 30–464 of HCMV gO. HEK293T cells were transiently transfected on 6-well plates with totally 6 µg DNA mixed with polyethyleneimine (Sigma). 48 h post transfection, cells were lysed for analysis of protein expression by WB or for immunoprecipitation experiments.

### Immunoprecipitation

Virions, transfected or infected cells or virus-cell co-incubations were lysed in standard lysis buffer (20 mM Tris-HCl, 150 mM NaCl, 1% Triton X-100, pH 8) containing cOmplete Mini protease inhibitor cocktail (Roche) for 1 h at 4°C. Lysates were cleared for 10 min at 18.000 x g at 4°C and then co-incubated with antibodies, recombinant PDGFRα-Fc or heparin agarose (Sigma) overnight at 4°C. The next day, Protein A Sepharose CL-4B (Avantor) was added to PDGFRα-Fc- or antibody-dependent co-incubations for another 4 h at 4°C. Beads were washed with lysis buffer and then 2x sample buffer (130 mM Tris-HCl, 10% glycerol, 10% 1-thioglycerol, 6% SDS, pH 6.8) was added to dissociate proteins from the beads. Alternatively, the precipitates were digested with sequencing grade modified porcine trypsin (Promega) overnight at 37°C followed by treatment with dithiothreitol (1 mM) for 10 min at 25°C, iodoacetamide (1.35 mM) for 30 min at 25°C and trifluoracetic acid (1.25%). The lyophilized samples were then analyzed by LC-MS/MS.

### Quantitative Fc pulldown assay

For comparing the binding of WT gO and gO249 protein to PDGFRα, WT and gO249 virions were lysed in standard lysis buffer. The gO protein content in lysates was adjusted for equal amounts. Aliquots of the lysates were co-incubated with increasing amounts of PDGFRα-Fc bound to Protein A sepharose beads. These beads were either produced by co-incubation with increasing amounts of PDGFRα-Fc (2–33 ng PDGFRα-Fc/µl beads) or by mixing PDGFRα-Fc-loaded beads (17 ng PDGFRα-Fc/µl beads) with PDGFRα-Fc-negative beads (6–50% positive beads). The co-precipitated gO proteins were analyzed by WB and signals quantified using ImageJ.

### Liquid chromatography tandem mass spectrometry (LC-MS/MS) analysis

LC-MS/MS analysis was performed using an Ultimate 3000 nano-liquid chromatography system (Thermo Fisher Scientific) coupled to a Q Exactive HF-X mass spectrometer (Thermo Fisher Scientific). Peptides were diluted in 0.1% formic acid and transferred to an Acclaim PepMap 100 trap column (nanoViper C18, 2 cm length, 100 μM ID, Thermo Fisher Scientific). An analytical EasySpray column (PepMap RSLC C18, 50 cm length, 75 μm ID, Thermo Fisher Scientific) was used for separation. Liquid chromatography was performed at a flow rate of 250 nl/min using 0.1% formic acid as solvent A and 0.1% formic acid in acetonitrile as solvent B. Peptides were eluted using an 80 min gradient from 5% to 20% solvent B, followed by a 10 min gradient from 20% to 40% B. Eluting peptides were analyzed using a data-independent acquisition method with 50 × 12 m/z wide precursor isolation windows in the range of 400–1000 m/z in a staggered window pattern. MS RAW data were processed using DIA-NN (v1.8) [50] with spectral libraries generated in DIA-NN by deep learning-based spectra and retention time prediction. As input sequence databases, the human subset of the UniProt database and a database consisting of the following HCMV proteins based on TB40-BAC4 were used: gH, gL, gO_WT_, gO_249_, UL128, UL130, UL131A and gB. In addition, the sequence of *Mus musculus* Ighg2b (UniProt: P01867) was implemented to allow quantification of the antibody as loading control of the immunoprecipitates. Volcano plot analysis was performed using Perseus (1.5.3.2) [51] with an two-tailed unpaired Student’s t-test and default values (FDR = 0.05, S0 = 0.1) as significance threshold. The mass spectrometry proteomics data have been deposited to the ProteomeXchange Consortium via the PRIDE [52] partner repository with the dataset identifier PXD058164.

### Western blot analysis

To analyze proteins in virus particles, cells or immunoprecipitates by WB, samples were lysed in 2x sample buffer. Proteins were subjected to SDS-polyacrylamide gel electrophoresis followed by WB analysis using nitrocellulose membranes for transfer of the proteins (0.45 µm, Thermo Fisher Scientific). Membranes were blocked with 5% skim milk and then incubated with primary and secondary antibodies. Antibody binding was detected using Super Signal West Pico chemiluminescence substrate (Thermo Fisher Scientific).

### Virion DNA isolation and qPCR

For viral DNA isolation from concentrated virus stocks or cell culture supernatants, DNA not protected in viral capsids was digested with 4 U DNAse I (NEB) for 1.5 h at 37°C. Then, 5 mM EDTA was added and the enzyme was heat-inactivated for 10 min at 75°C.

Viral DNA in particles was isolated using the DNeasy Blood & Tissue Kit (QIAGEN) according to the manufacturer’s protocol. Briefly, the DNAse I-digested samples or virions attached to cells were incubated with Proteinase K in lysis buffer for 10 min at 56°C followed by the addition of ethanol. Then, the samples were transferred onto the provided columns, washed and the extracted DNA was eluted in DNAse and RNAse free ddH_2_O.

To quantify the amount of virion DNA, qPCR was performed. A reaction mixture of 20 µl containing 1x SYBR Green PCR Master Mix (Applied Biosystems), 2 µl isolated virion DNA and 500 nM of UL83 forward (5’-TGGTCACCTATCACCTGCAT-3’) and reverse (5’-GAAAGAGCCCGACGTCTACT-3’) primer was prepared. DNA was detected using the QuantStudio 5 Real-Time-PCR-System (Applied Biosystems). To calculate the number of viral genomes (copies/ml), a TB40-BAC4 bacmid standard was used.

### Heparinase digestion

To remove cell surface HSPGs, HFF or TIME cells were incubated for 2 h at 30°C with heparinase I, II and III (1 U/ml, NEB) in Opti-PRO medium (Gibco) supplemented with 25 mM HEPES (Gibco). Excess heparinases were removed by washing the cell monolayers with DPBS.

### Luciferase assay

Luciferase assays were performed as described previously [8]. Briefly, HFF or TIME cells were seeded on 96-well plates and infected the next day. If inhibition assays were performed, PDGFRα-Fc or heparin were serially diluted and mixed with the respective viruses. For PDGFRα-Fc inhibition, the virus – PDGFRα mixtures were pre-incubated for 1 h on ice before virus was added. For inhibition assays with recombinant gH_Strep_gLgO, the protein was serially diluted and co-incubated with the cells for 1 h at 37°C followed by the addition of the virus. For infection, a centrifugation step for 30 min at 860 x g at RT was performed followed by co-incubation of the cells and viruses for 1.5 h at 37°C. Then, the supernatants were exchanged for medium containing phosphonoacetic acid (300 µg/ml, Sigma). 48 h post-infection, the luciferase activity in infected cells was determined using the Firefly Luciferase Assay Kit 2.0 (Biotium) according to the manufacturer’s protocol.

### Binding assay

HFF or TIME cells were seeded in 24-well plates. The next day, the cells were pre-cooled for 10 min on ice before virus particles were added in a volume of 250 µl medium. To inhibit binding to HSPGs, heparin (100 µg/ml) was added to the medium or the cells were pre-incubated with heparinases before addition of virus. Binding was allowed for 1 h on ice followed by three washing steps on ice to remove unbound virus. Finally, DNA was extracted from the cell monolayers using the DNeasy Blood & Tissue Kit and qPCR was performed to determine the number of virion particles bound to the cells.

### ELISA for gH_strep_gLgO quantification

HFF or TIME cells were seeded on 96 well plates. The next day, cells were incubated with recombinant HCMV gH_Strep_gLgO (100 µg/ml) for 1 h at 37°C. After removal of unbound protein, the cells were fixed with 1% PFA for 10 min at RT. To detect gH_Strep_gLgO_WT_ complexes bound to cell surfaces, cells were incubated with mouse anti-Strep antibody for 1 h at RT followed by incubation with peroxidase-coupled goat anti-mouse antibody (1:2000) for 1 h at RT. Then, TMB substrate (BioLegend) was added onto the cells. The reaction was stopped with 10% phosphoric acid and the absorbance determined at 450 nm.

### Statistical analysis

Data were analyzed and statistical significance was determined with GraphPad Prism 10.1.2. Means of two groups were compared by two-tailed unpaired Student’s t-test. Means of multiple groups were compared by ordinary one-way ANOVA with Turkey’s multiple comparisons test with a single pooled variance. If data were not normally distributed, Student’s t-test was replaced by a two-tailed Mann-Whitney test and one-way ANOVA was replaced by a Kruskal-Wallis test with Dunn’s multiple comparisons.

### Data visualization and quantification

Structures were visualized using PyMOL 2.5.5. Schematic presentations were created with BioRender and Western blots were quantified with ImageJ 1.52a.

## Supporting information

S1 Fig Complementary characterization of cells and gO mutants. (**a**) Endogenous PDGFRα expression levels in HFF and TIME cells were determined by WB analysis of total cell lysates. GAPDH served as a loading control. (**b**) Multistep growth curves of different gO mutants on HFF. Infectious supernatant virus was determined by a TCID50 assay. Right panel: Particle numbers quantified by qPCR and specific infectivity of virus particles determined for day eight growth curve supernatants.
(TIF)

S2 Fig Binding of wildtype gO and gO249 to PDGFRα. (**a**) Levels of virion glycoproteins gH, gO and gB were assessed by WB analysis in lysates of WT, gO249, 131stop and 131stop-gO249 virions. MCP levels reflect comparable numbers of virus particles. One representative experiment is shown. (**b**) Quantification of gH and gO in 131stop and 131stop-gO249 virion lysates by WB. Left panel: Two exposures of a representative WB. Right panel: ImageJ quantification of the short exposure. The peak areas of the WB signals for gH and gO are indicated. (**c**) Quantitative Fc pulldown assay to determine binding curves for wildtype gO and gO249. Top: ImageJ quantification of the WB signal of gO precipitated by increasing amounts of PDGFRα-Fc. Bottom: Depiction of the original WBs. Two independent approaches were used to produce increasing amounts of PDGFRα-Fc bound to beads. Left panel: Increasing amounts of PDGFRα-Fc per µl beads during coating of beads. Right panel: Percentage of precoated PDGFRα-Fc beads of the total amount of beads used. One representative experiment out of two (left) or three (right) is shown. (**d**) WB analysis of gH and gO precipitated from lysates of HEK293T cells expressing gHgL, gHgLgO_WT_ or gHgLgO_249_ using recombinant PDGFRα-Fc. GAPDH levels served as loading control. One experiment of two is shown.
(TIF)

S3 Fig Screening for gHgLgO interaction partners on TIME cells. Volcano plot of LC-MS/MS data from anti-gH immunoprecipitates of lysates of TIME cells co-incubated with 131stop or 131stop-gO249 virions as described in Fig 2a. Data from 131stop-gO249 virions were compared to 131stop virions and depicted as –Log p-value versus Log2-fold change. HCMV glycoproteins are highlighted in red and TGFβRIII in blue. Data are derived from three independent experiments.
(TIF)

S4 Fig Analysis of gH and gO expressed in infected HFF and virions. WB analysis of gH and gO in lysates of HFF infected with WT or gO249 virus and in lysates of the respective virions. MCP and GAPDH expression levels served as loading controls. One representative experiment of three is shown.
(TIF)

S5 Fig Blocking of HFF and TIME cell infection by recombinant gH_Strep_gLgO. Infection of (**a**) HFF or (**b**) TIME cells after preincubation of the cells with increasing concentrations of recombinant gH_Strep_gLgO. 48 h p.i., infection was assessed by luciferase assay. Shown are means + /- SD of one experiment performed in triplicates.
(TIF)

S6 Fig A role of gB in HSPG-dependent tethering. (**a**) WB analysis of gH, gO, gB and UL128 precipitated from lysates of WT or ΔgO virions using heparin agarose. One representative experiment of four is shown. (**b**) HFF (*n*(WT, gO249) = 6, *n*(ΔgO) = 3) or (**c**) TIME cells (*n *= 3) were co-incubated with 5x10^7^ WT, gO249 or ΔgO virus particles in the presence (100µg/ml) or absence of heparin and virus particles bound to cells were quantified by qPCR. Shown are means + /- SEM of independent experiments. Statistical significance was determined for pairwise comparisons of heparin-treated or untreated infections (Student’s t-test). Additionally, fold changes were calculated and analyzed (one-way ANOVA). *P* values of statistically significant differences are depicted. The data showing virus attachment of WT and gO249 virions both in the presence and absence of heparin are identical to the data of Fig 3e and 3f.
(TIF)

S1 Table LC-MS/MS hits of interaction partners (HFF) of 131stop-gO249 versus 131stop virions. LC-MS/MS data from anti-gH (14-4b) immunoprecipitates of lysates of HFF co-incubated with 131stop or 131stop-gO249 virions. Data correspond to the Volcano plot shown in Fig 2c and are depicted as fold change 131stop-gO249 virus versus 131stop virus. Additionally, the respective *P* values are shown. Selected proteins and proteoglycans are highlighted (HCMV glycoproteins (red), PDGFRα (green), TGFβRIII, syndecans and glypican (blue)).
(TIF)

S2 Table LC-MS/MS hits of interaction partners (TIME cells) of 131stop-gO249 versus 131stop virions. LC-MS/MS data from anti-gH (14-4b) immunoprecipitates of lysates of TIME cells co-incubated with 131stop or 131stop-gO249 virions. Data correspond to the Volcano plot shown in S3 Fig and are depicted as fold change 131stop-gO249 virus versus 131stop virus. Additionally, the respective *P* values are shown. Selected proteins are highlighted (HCMV glycoproteins (red), TGFβRIII (blue)). A list of all detected hits from this LC-MS/MS analysis is provided in S3 Table.
(TIF)

S3 Table Comprehensive list of LC-MS/MS hits of immunoprecipitates of lysates of TIME cells co-incubated with 131stop or 131stop-gO249 virions. Conditions (cell control, 131stop, 131stop-gO249) and respective biological replicates are indicated (replicates 1–3).
(XLSX)
