# Supplementary figures and images for: Identification of the human cytomegalovirus gHgLgO trimer as the central player in virion infectivity
Source: PLoS Pathog. 2025 Jul 24;21(7):e1013341. doi: 10.1371/journal.ppat.1013341 (PMC12316387; doi:10.1371/journal.ppat.1013341)

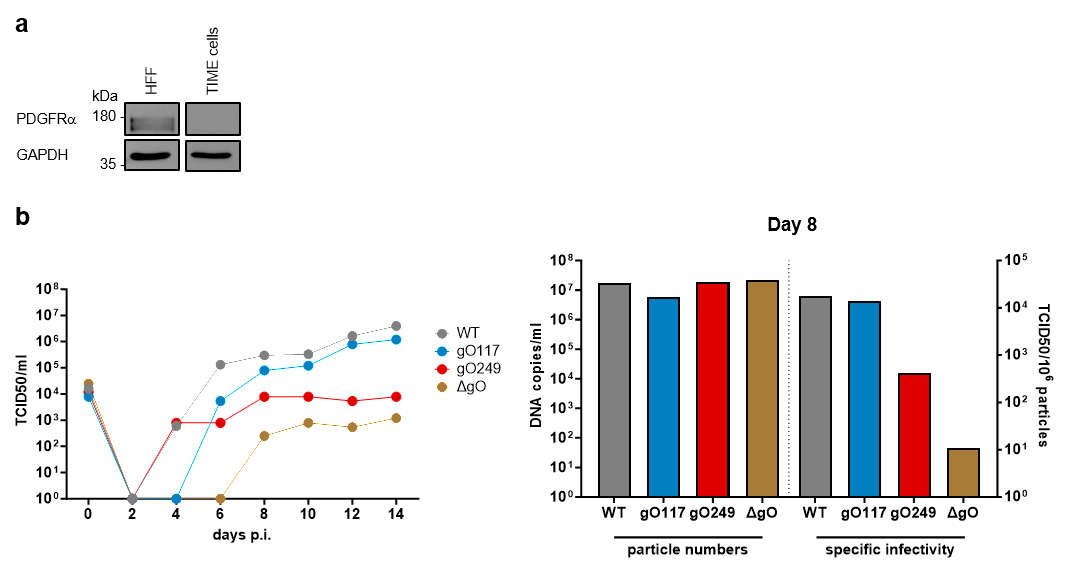

Supplement: S1 Fig — (a) Endogenous PDGFRα expression levels in HFF and TIME cells were determined by WB analysis of total cell lysates. GAPDH served as a loading control. (b) Multistep growth curves of different gO mutants on HFF. Infectious supernatant virus was determined by a TCID50 assay. Right panel: Particle numbers quantified by qPCR and specific infectivity of virus particles determined for day eight growth curve supernatants. (TIF) [file ppat.1013341.s001.tif]

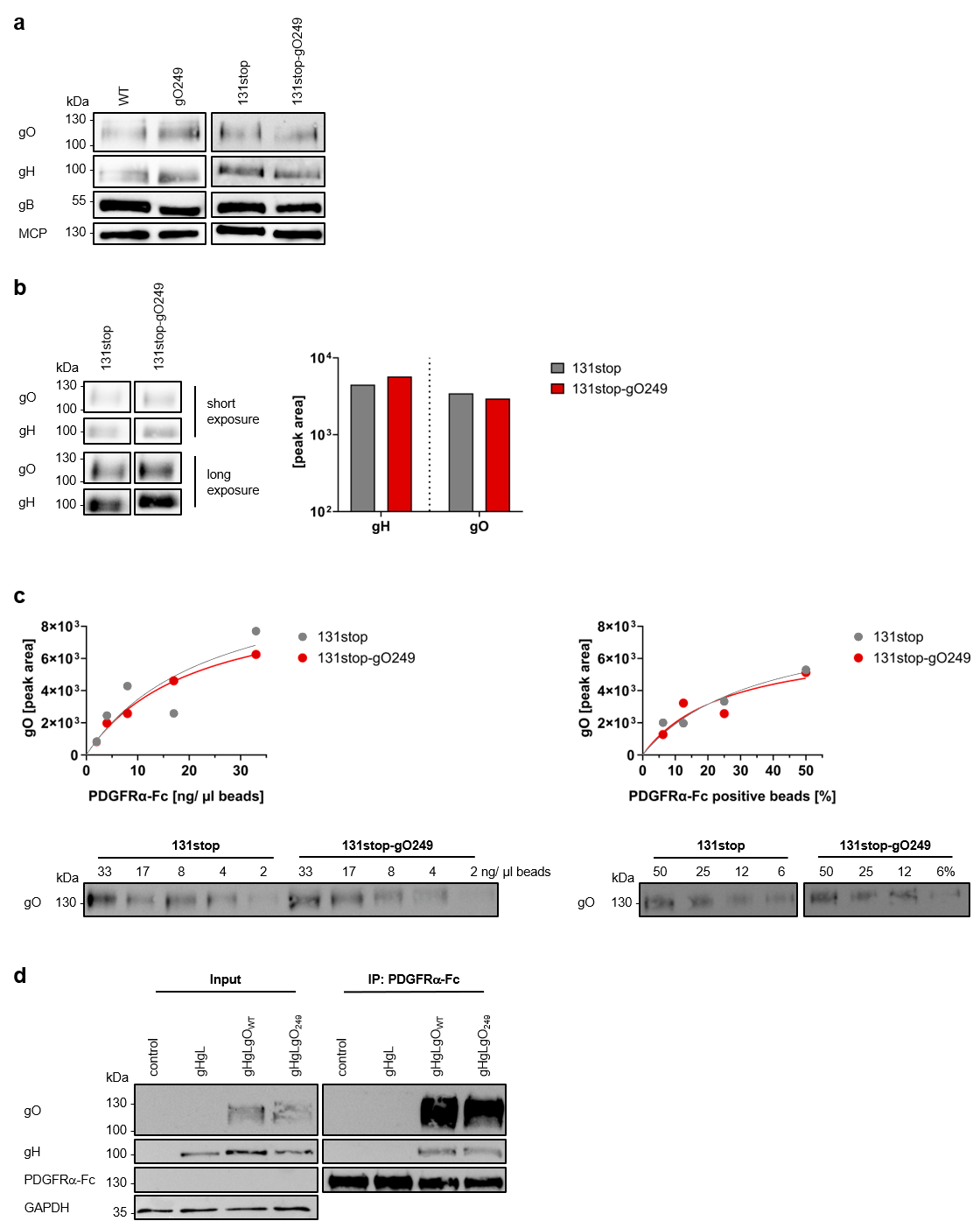

Supplement: S2 Fig — (a) Levels of virion glycoproteins gH, gO and gB were assessed by WB analysis in lysates of WT, gO249, 131stop and 131stop-gO249 virions. MCP levels reflect comparable numbers of virus particles. One representative experiment is shown. (b) Quantification of gH and gO in 131stop and 131stop-gO249 virion lysates by WB. Left panel: Two exposures of a representative WB. Right panel: ImageJ quantification of the short exposure. The peak areas of the WB signals for gH and gO are indicated. (c) Quantitative Fc pulldown assay to determine binding curves for wildtype gO and gO249. Top: ImageJ quantification of the WB signal of gO precipitated by increasing amounts of PDGFRα-Fc. Bottom: Depiction of the original WBs. Two independent approaches were used to produce increasing amounts of PDGFRα-Fc bound to beads. Left panel: Increasing amounts of PDGFRα-Fc per µl beads during coating of beads. Right panel: Percentage of precoated PDGFRα-Fc beads of the total amount of beads used. One representative experiment out of two (left) or three (right) is shown. (d) WB analysis of gH and gO precipitated from lysates of HEK293T cells expressing gHgL, gHgLgOWT or gHgLgO249 using recombinant PDGFRα-Fc. GAPDH levels served as loading control. One experiment of two is shown. (TIF) [file ppat.1013341.s002.tif]

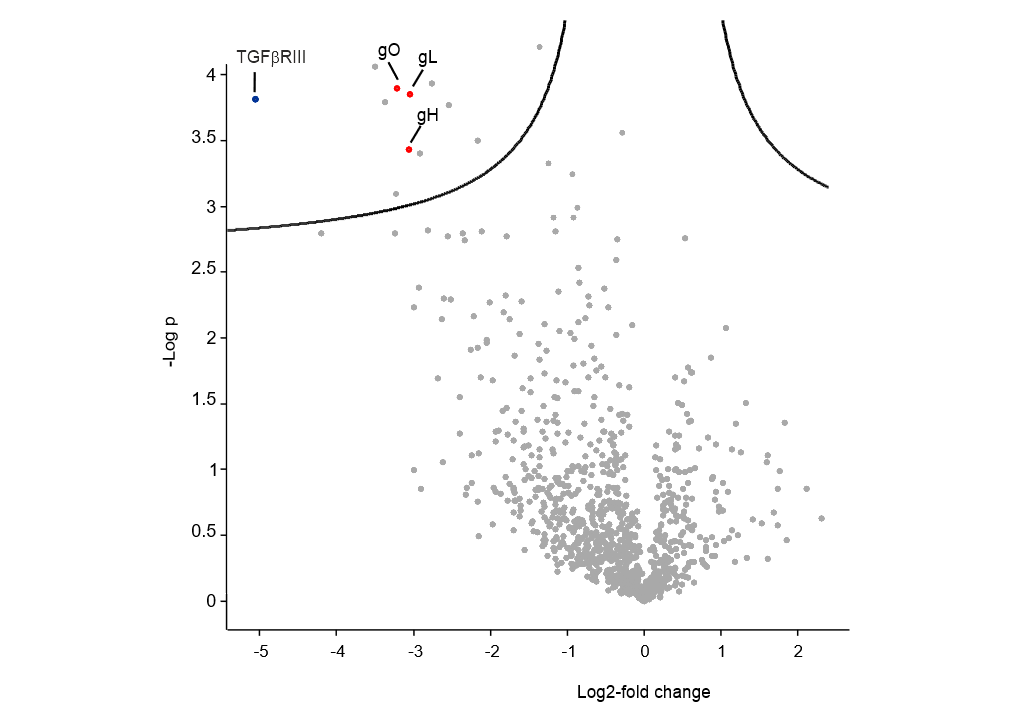

Supplement: S3 Fig — Volcano plot of LC-MS/MS data from anti-gH immunoprecipitates of lysates of TIME cells co-incubated with 131stop or 131stop-gO249 virions as described in Fig 2a. Data from 131stop-gO249 virions were compared to 131stop virions and depicted as –Log p-value versus Log2-fold change. HCMV glycoproteins are highlighted in red and TGFβRIII in blue. Data are derived from three independent experiments. (TIF) [file ppat.1013341.s003.tif]

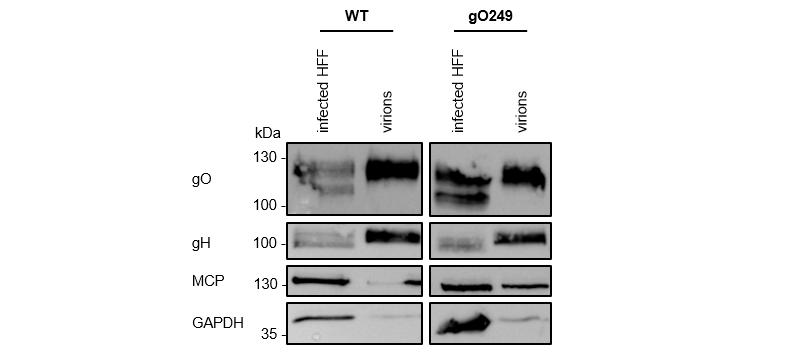

Supplement: S4 Fig — WB analysis of gH and gO in lysates of HFF infected with WT or gO249 virus and in lysates of the respective virions. MCP and GAPDH expression levels served as loading controls. One representative experiment of three is shown. (TIF) [file ppat.1013341.s004.tif]

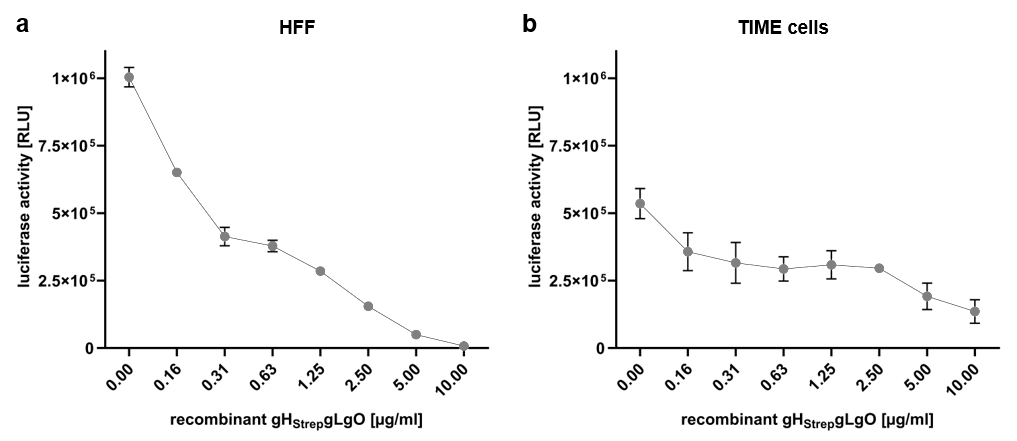

Supplement: S5 Fig — Infection of (a) HFF or (b) TIME cells after preincubation of the cells with increasing concentrations of recombinant gHStrepgLgO. 48 h p.i., infection was assessed by luciferase assay. Shown are means + /- SD of one experiment performed in triplicates. (TIF) [file ppat.1013341.s005.tif]

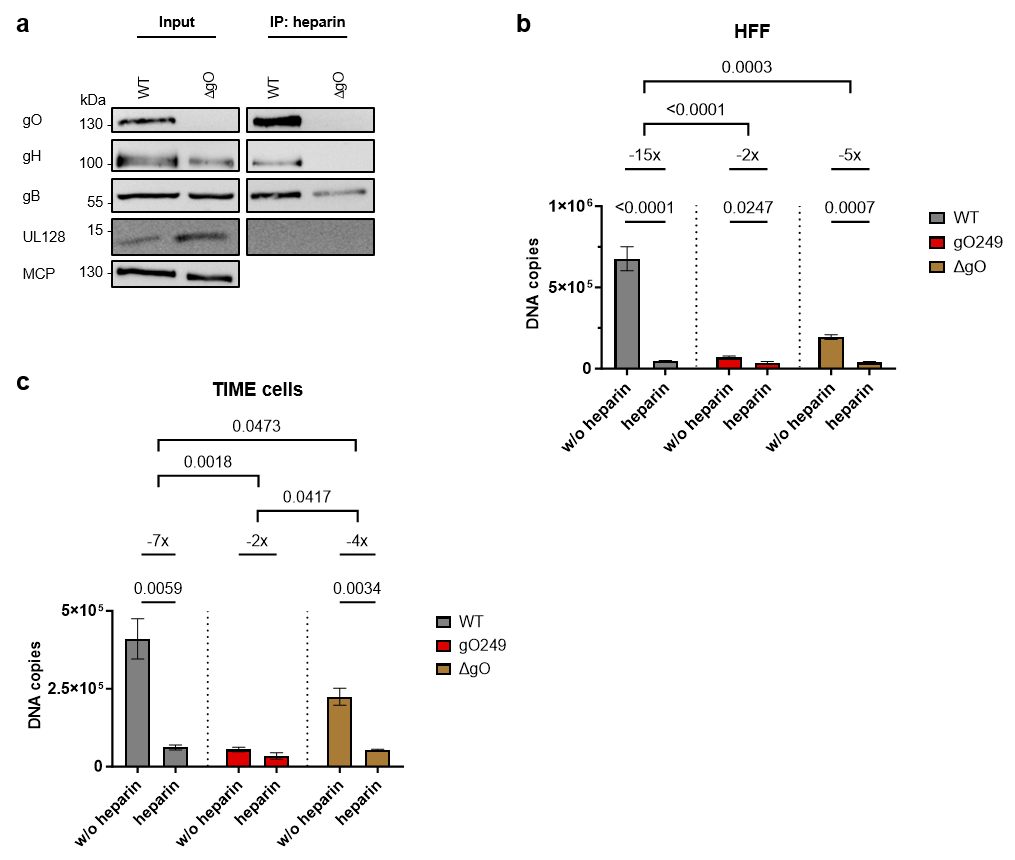

Supplement: S6 Fig — (a) WB analysis of gH, gO, gB and UL128 precipitated from lysates of WT or ΔgO virions using heparin agarose. One representative experiment of four is shown. (b) HFF (n(WT, gO249) = 6, n(ΔgO) = 3) or (c) TIME cells (n = 3) were co-incubated with 5x10^7^ WT, gO249 or ΔgO virus particles in the presence (100µg/ml) or absence of heparin and virus particles bound to cells were quantified by qPCR. Shown are means + /- SEM of independent experiments. Statistical significance was determined for pairwise comparisons of heparin-treated or untreated infections (Student’s t-test). Additionally, fold changes were calculated and analyzed (one-way ANOVA). P values of statistically significant differences are depicted. The data showing virus attachment of WT and gO249 virions both in the presence and absence of heparin are identical to the data of Fig 3e and 3f. (TIF) [file ppat.1013341.s006.tif]

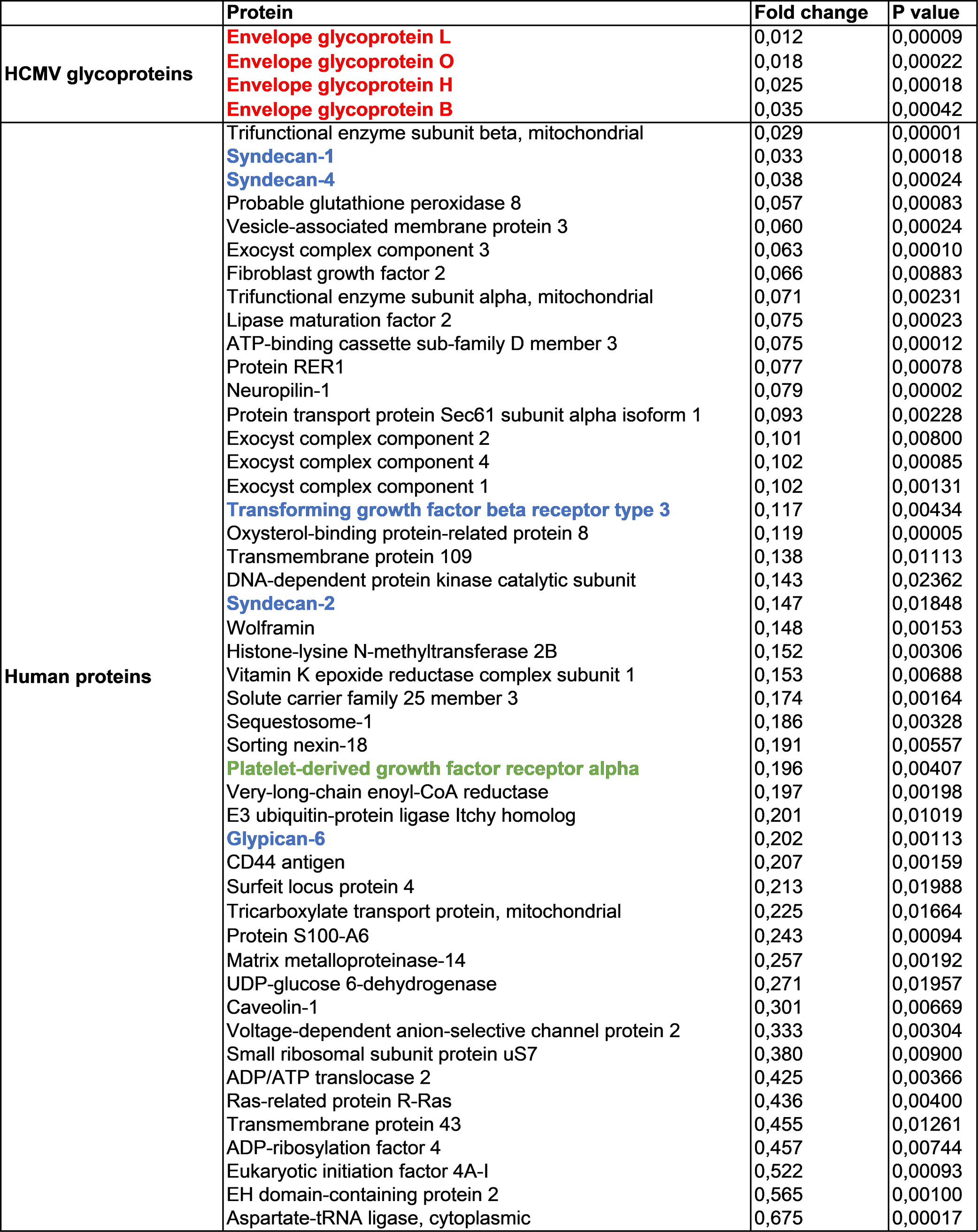

Supplement: S1 Table — LC-MS/MS data from anti-gH (14-4b) immunoprecipitates of lysates of HFF co-incubated with 131stop or 131stop-gO249 virions. Data correspond to the Volcano plot shown in Fig 2c and are depicted as fold change 131stop-gO249 virus versus 131stop virus. Additionally, the respective P values are shown. Selected proteins and proteoglycans are highlighted (HCMV glycoproteins (red), PDGFRα (green), TGFβRIII, syndecans and glypican (blue)). (TIF) [file ppat.1013341.s007.tif]

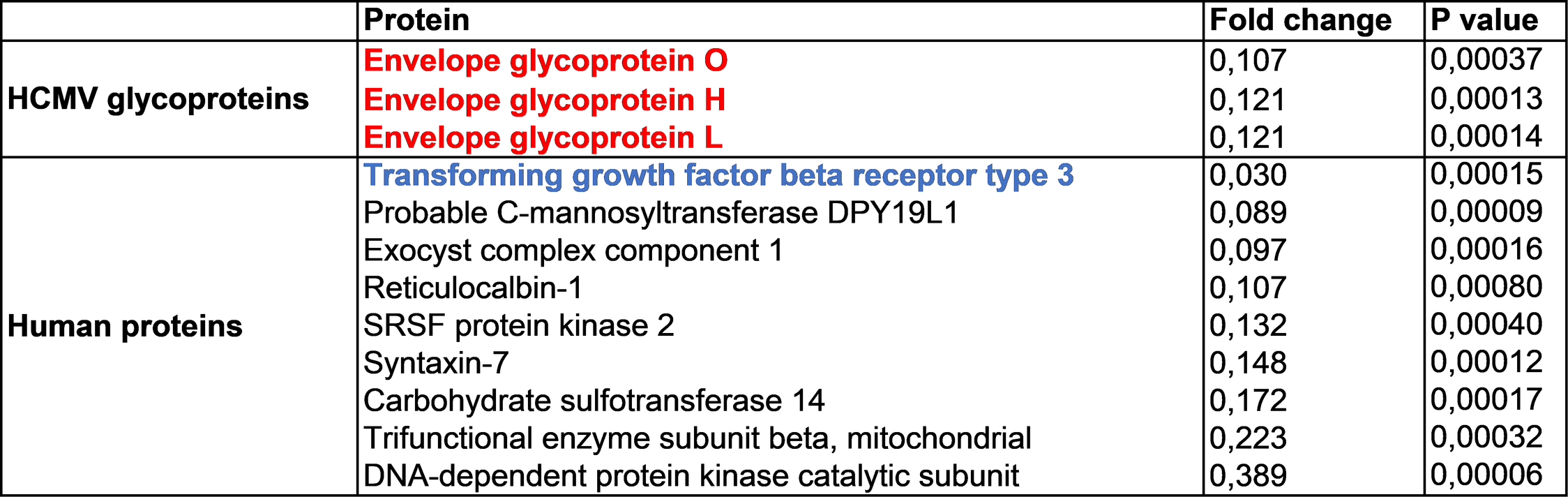

Supplement: S2 Table — LC-MS/MS data from anti-gH (14-4b) immunoprecipitates of lysates of TIME cells co-incubated with 131stop or 131stop-gO249 virions. Data correspond to the Volcano plot shown in S3 Fig and are depicted as fold change 131stop-gO249 virus versus 131stop virus. Additionally, the respective P values are shown. Selected proteins are highlighted (HCMV glycoproteins (red), TGFβRIII (blue)). A list of all detected hits from this LC-MS/MS analysis is provided in S3 Table. (TIF) [file ppat.1013341.s008.tif]
